# Assessment of the bony resection margin distance in bone-invasive oral cancer using laser-induced breakdown spectroscopy

**DOI:** 10.1007/s00784-024-05862-5

**Published:** 2024-08-08

**Authors:** Philipp Winnand, Mark Ooms, Marius Heitzer, Nils Vohl, Matthias Lammert, Frank Hölzle, K. Olaf Boernsen, Ali Modabber

**Affiliations:** 1 Department of Oral and Maxillofacial Surgery, University Hospital RWTH Aachen, Pauwelsstraße 30, 52074 Aachen, Germany; 2 Institute of Pathology, University Hospital RWTH Aachen, Pauwelsstraße 30, 52074 Aachen, Germany; 3 Institute of Chemistry and Bioanalytics, University of Applied Sciences Northwestern Switzerland, Hofackerstrasse 30, CH-4132 Muttenz, Switzerland

**Keywords:** Bone-invasive oral cancer, Bone tumor resection margins, Laser-induced breakdown spectroscopy, Rapid bone analysis

## Abstract

**Objectives:**

Inadequate resection margins of less than 5 mm impair local tumor control. This weak point in oncological safety is exacerbated in bone-infiltrating tumors because rapid bone analysis procedures do not exist. This study aims to assess the bony resection margin status of bone-invasive oral cancer using laser-induced breakdown spectroscopy (LIBS).

**Materials and methods:**

LIBS experiments were performed on natively lasered, tumor-infiltrated mandibular cross-sections from 10 patients. In total, 5,336 spectra were recorded at defined distances from the tumor border. Resection margins < 1 mm were defined as very close, from 1–5 mm as close, and > 5 mm as clear. The spectra were histologically validated. Based on the LIBS spectra, the discriminatory power of potassium (K) and soluble calcium (Ca) between bone-infiltrating tumor tissue and very close, close, and clear resection margins was determined.

**Results:**

LIBS-derived electrolyte emission values of K and soluble Ca as well as histological parameters for bone neogenesis/fibrosis and lymphocyte/macrophage infiltrates differ significantly between bone-infiltrating tumor tissue spectra and healthy bone spectra from very close, close, and clear resection margins (*p* < 0.0001). Using LIBS, the transition from very close resection margins to bone-infiltrating tumor tissue can be determined with a sensitivity of 95.0%, and the transition from clear to close resection margins can be determined with a sensitivity of 85.3%.

**Conclusions:**

LIBS can reliably determine the boundary of bone-infiltrating tumors and might provide an orientation for determining a clear resection margin.

**Clinical relevance:**

LIBS could facilitate intraoperative decision-making and avoid inadequate resection margins in bone-invasive oral cancer.

## Introduction

Surgery is an integral part of the treatment of resectable head and neck cancers [1, 2]. Resection margin distances are defined as clear for > 5 mm, close for 1–5 mm, and positive for < 1 mm [3]. In a slight modification of this classification, only resection margins that reach the tumor are described as positive, so tumor-free resection margins with a distance of < 1 mm from the tumor border are described as very close [4]. The presence of adequate resection margins significantly influences survival in oral cancer, with 5-year survival rates of 81% for clear, 75% for close, and 54% for positive resection margin status [5]. With an incidence of inadequate (close and positive) resection margins of 30–65%, locoregional failure constitutes a major problem after oral cancer resection [6].

In general, adequate tumor resection requires the surgeon to perform a correct assessment of the tumor boundary. However, macroscopic definition of the tumor margins by visual inspection and palpation may fail to detect phenomena like microscopic disease beyond the gross tumor mass [7, 8]. In the case of bone-infiltrating tumors, intraoperative estimation of the tumor border is further complicated by the absence of options for securing tumor-free resection margins in the bone [8, 9]. Although the absence of a rapid analysis of bone is not an exclusive characteristic of oral cancer, it must be given a game-changing role in the context of the resection of bone-invasive tumors of the oral cavity [10]. Segmental mandibulectomy overestimates the bony resection margins in 16% [9] to 43% [11] of all cases, complicating the patient’s masticatory, respiratory, and speech functional rehabilitation. In contrast, intraoperative failure to achieve tumor-free bone margins, the incidence of which has been reported to be around 10% [12, 13], results in a local tumor control loss [8, 14] and a worsening of the patients’ prognosis [9, 15].

As the current state of the art does not permit intraoperative reresection of positive bone margins, the absence of rapid bone analytical diagnostics must be compensated for to prevent local recurrence in bone. Thus, the spatial complexity of bony tumor resection margins can be anticipated by increasing the radicality and extending the resection margin distance in bone to 15 mm from the macroscopic tumor margin [8]. Alternatively or additionally, sampling of cortical bone scrapings, cancellous bone and the inferior alveolar nerve may provide additional intraoperative orientation [16].

Contactless detection of bony invasive growth of head and neck tumors using single imaging modalities is limited [17]. Spectroscopic techniques can be used to objectively visualize the tumor boundary [18, 19]. As a technique for direct analysis, laser-induced breakdown spectroscopy (LIBS) can discriminate the tumor boundary in brain tumors [20], lung cancers [21], and cutaneous melanoma [22, 23]. The technical components of a LIBS-based optical scanner can be optimized for the robust analysis of native sample material to meet validated biomarker assays’ criteria [24]. Based on this, the possibility of bony rapid analysis with LIBS was evaluated, which showed significant differences between healthy bone tissue and bone-infiltrating oral tumor tissue based on the electrolyte extinction values of potassium (K), calcium (Ca), and sodium (Na) [25]. Depth profiles of spectra and electrolytes are useful for detecting the microscopic spread of oral cancer in bone, which indicates that the complexity of the tumor boundary in oral cancer with bony invasion can be reduced with LIBS [26]. In addition, LIBS can be used to assess the electrolyte heterogeneity of nontumorous and tumorous tissues in bone-infiltrating oral cancers [27].

Continuing our research efforts, our previously trained tissue recognition algorithms [25, 27] will be tested to identify the tumor boundary in bone-infiltrative oral cavity cancer and assess the margin status of bony resection with a LIBS-based focus on electrolyte distribution.

## Material and methods

### Ethical approval statement

Ethical approval was obtained from the Medical Faculty of the RWTH Aachen University, Germany (EK 126/21). Mandibular bone sections were collected via the RWTH Centralized Biomaterial Bank (RWTH cBMB) from patients who had previously given written informed consent. The study was performed following the Declaration of Helsinki.

### Protocol for mandibular cross-sections

Segmental mandibular resection specimens from 10 patients with bone-invasive oral cancer were included in the study sample. After a joint macroscopic assessment by a surgeon and a pathologist, the segmental mandibular resection specimen was sawn into 4 mm thick slices in its native state. Mandible bone slices that were not necessary for routine histology procedures were frozen in their native state at -20 °C and stored until LIBS instrumentation. Immediately before the start of the LIBS experiments, the bone slices were thawed, rinsed with NaCl 0.9%, and dried with sterile compresses.

### LIBS instrumentation and mapping

The Advanced Osteotomy Tools (AOT) experimental LIBS setup is based on a Q-switched Nd:YAG laser with a wavelength of 1,064 nm. Thirty laser shots were applicated at each position. The experimental setup was described in detail in our previous publications [24–27].

A thin separating disc was used to transfer a two-dimensional coordinate system to the bone slices. Vertical and horizontal markings were milled at 1 mm intervals. LIBS spectra were acquired along the specified coordinates starting from the bone and moving toward the bone-infiltrating tumor tissue. The acquisition of the LIBS spectra took 30 min per mandible section.

### Histological annotation and histological analysis

**Fig. 1:**
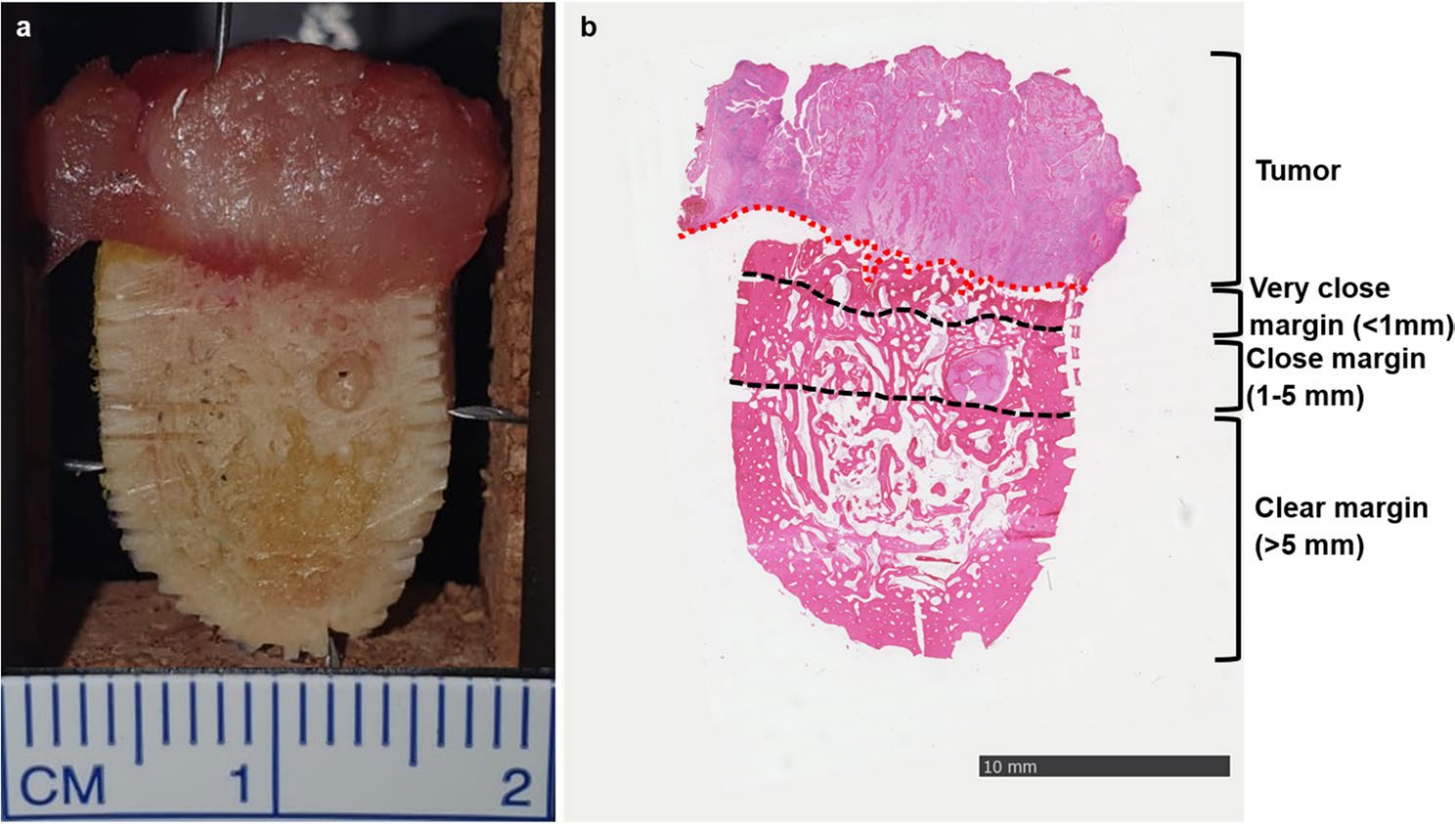
Native (**a**) and HE-stained mandible (**b**) bone section with bone-invasive oral cancer and very close (< 1 mm), close (1–5 mm), and clear (> 5 mm) resection margin areas. The tumor border is marked in red

**Fig. 2:**
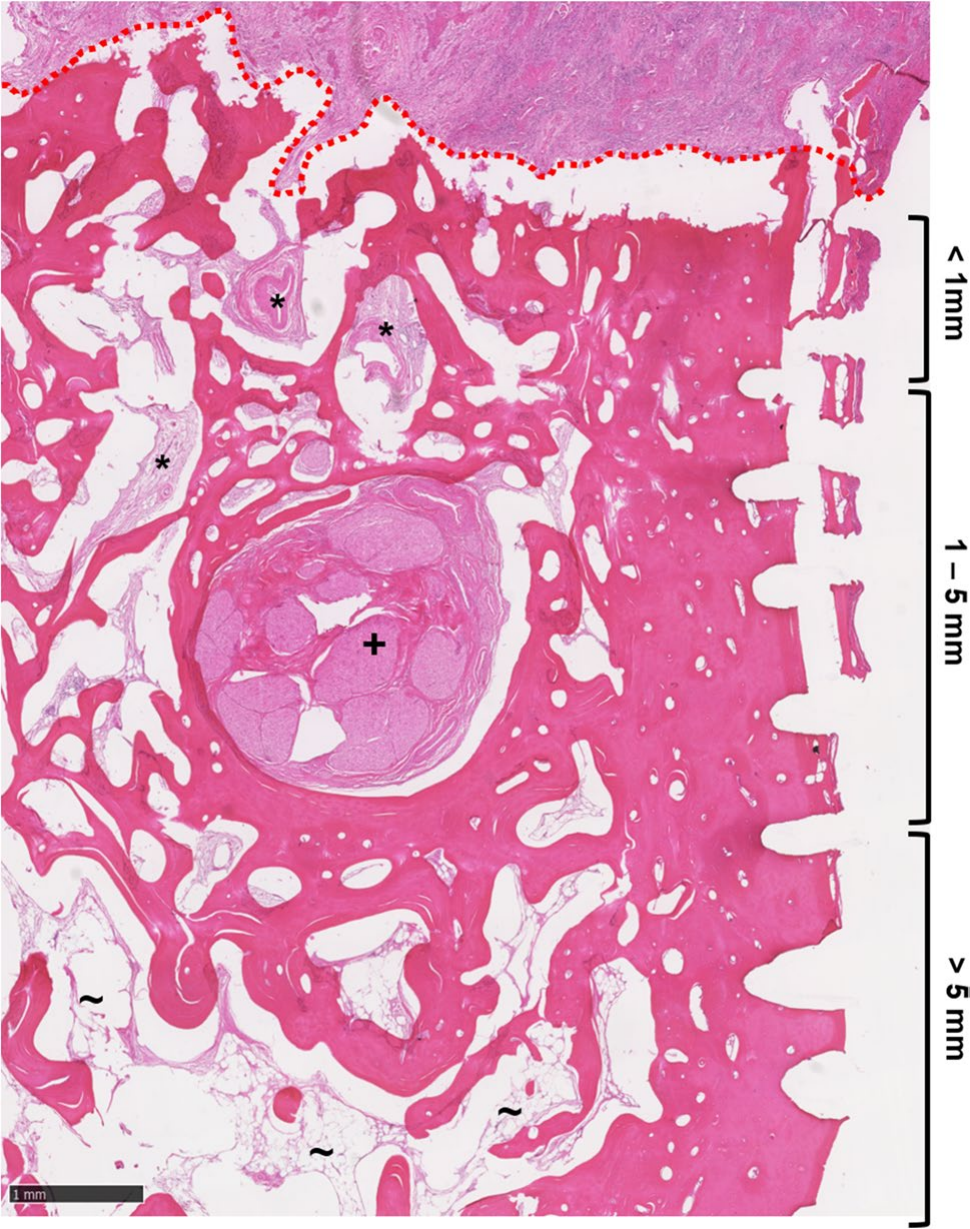
HE section (2.5 × magnification) with fibroinflammatory reaction (*) of the bone in the very close and close margin area and fatty bone marrow spaces (**~**) in the clear margin area. Note the inferior alveolar nerve (**+**) location in the close margin area. The tumor border is marked in red

Following the LIBS experiments, the mandible sections were subjected to routine histology. This included decalcification in ethylenediamine tetraacetic acid (EDTA), embedding in paraffin, cutting, and hematoxylin and eosin (H&E) staining. Vertical and horizontal markings were used to assign the individual spectra of the bone slices to the corresponding localizations. The tumor border was histologically validated (Figs. 1 and 2). The LIBS spectra were sorted according to the distance to the tumor border so that LIBS spectra from the bone-infiltrating tumor tissue, from very close (< 1 mm), close (1–5 mm), and clear (> 5 mm) resection margin distances, were available for further analysis.

Each laser spot position (diameter: 240 μm) included in the final analysis was histologically evaluated according to the chronic osteomyelitis criteria of the histopathological osteomyelitis evaluation score (HOES) according to Tiemann et al. [28]. A dedicated pathologist performed a semiquantitative evaluation of bone neogenesis/fibrosis and lymphocyte/macrophage infiltrates of each laser spot position using a scale of 0 (nonexistent), 1 (mild = one-third of the section area), 2 (moderate = two-thirds of the section area), and 3 (severe = three-third of the section area).

### Processing of the spectra and peak area calculations

No averaging was used when recording the LIBS spectra. According to AOT’s data processing method, the baseline of the spectrometer was deducted, and the spectra were normalized to the intensity of the baseline peak. As previously described [24–27], the spectra were analyzed using peak area calculation. Soluble Ca was measured at 547 nm, 596 nm, and 610 nm, while K was measured at 765 nm and 770 nm. For further processing and spectral analysis, Microsoft Excel and GraphPad Prism 10 were used.

### Statistical analysis

Electrolyte emission values were reported as median values with interquartile ranges. Histological parameters were reported as numbers with percentages. Differences in electrolyte emission values and histological parameters between bone-infiltrating tumor tissue and tissues representing very close, close, and clear margins were tested using the Kruskal–Wallis test. Pairwise comparison in the case of significant global testing was performed using Dunn’s post hoc test with p-values adjusted for multiple testing.

Trends in progression from very close margin to tumor, close margin to very close margin, and clear margin to close margin were analyzed using a logistic regression analysis separately for K, soluble Ca, and soluble Ca/K ratio, adjusted for age, gender, and G-status.

Receiver operating characteristics (ROC) analyses were performed for K and soluble Ca/K ratio for detection of resection margin status (very close vs. tumor, close vs. very close, and clear vs. close) [29]. The theoretical optimal threshold value for the detection of resection margin status was determined by calculation of the Youden index [30]. Diagnostic accuracy was analyzed by calculating the area under the curve [29].

Statistical significance was assumed for p-values < 0.05. Statistical analysis was executed using SPSS version 28. Visualization of electrolyte differences between bone-infiltrating tumor tissue and tissues representing very close, close, and clear resection margins was performed with GraphPad Prism 10.

## Results

### Comparison of electrolyte emission values in bone-infiltrating tumor tissue and very close, close, and clear resection margins

**Fig. 3:**
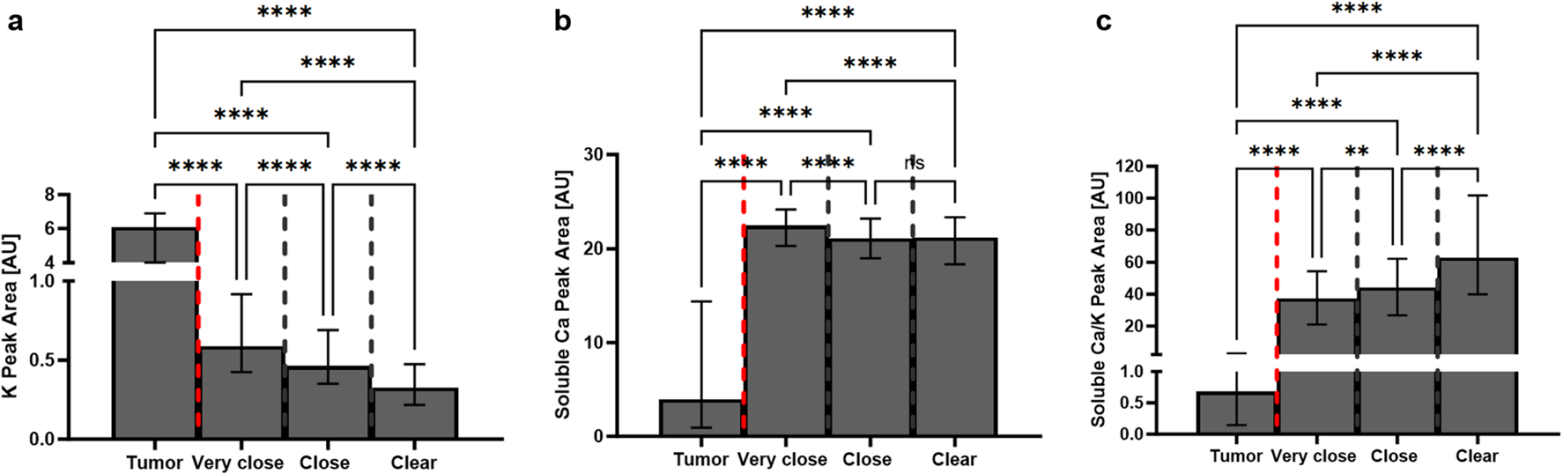
Bar column shown as the median with interquartile range of K (**a**) and soluble Ca (**b**) extinction values, and soluble Ca/K ratios (**c**) in tumor, very close (< 1 mm), close (1–5 mm), and clear (> 5 mm) resection margin areas

The emission values of K and soluble Ca, and the soluble Ca/K ratio differed significantly (*p* < 0.0001) between bone-infiltrating tumor tissue and bone spectra from very close, close, and clear resection margins (Table 1). Emission levels of K were significantly (*p* < 0.0001) higher in bone-infiltrating tumor tissue than in very close, close, and clear bony resection margins. Bone-infiltrating tumor tissue contained significantly (*p* < 0.0001) less soluble Ca than very close, close, and clear resection margins. The soluble Ca/K ratio was significantly (*p* < 0.0001) lower in bone-infiltrating tumor tissue than in very close, close, and clear bony resection margins. Further details are shown in Fig. 3.

**Table 1:** Comparison of electrolyte emission values between very close, close, and clear margins in bone substance and bone-infiltrating tumor tissue

*Variable*	*Tumor* *(n* = *1298 spectra)*	*Very close margin* *(n* = *336 spectra)*	*Close margin (n* = *1092 spectra)*	*Clear margin* *(n* = *2610 spectra)*	*p value*
*K*	6.079^1; 2; 3^	0.587^2; 3; 4^	0.463^1; 3; 4^	0.326^1; 2; 4^	**< 0.0001**
*Soluble Ca*	3.968^1; 2; 3^	22.422^2; 3; 4^	21.044^1; 4^	21.153^1; 4^	**< 0.0001**
Soluble Ca/K	0.681^1; 2; 3^	37.070^2; 3; 4^	44.340^1; 3; 4^	62.782^1; 2; 4^	**< 0.0001**

The parameters are shown as median values. The differences between the parameters were tested with the Kruskal–Wallis test and with the Dunn’s post hoc test which was used for pairwise testing. Significant p values are bold. 1: *p* < 0.05 vs. very close margin. 2: *p* < 0.05 vs. close margin. 3: *p* < 0.05 vs. clear margin. 4: *p* < 0.05 vs. tumor

### Comparison of histological parameters in bone-infiltrating tumor tissue and very close, close, and clear resection margins

Histological parameters of bone neogenesis and fibrosis were significantly (*p* < 0.0001) different between bone-infiltrating tumor tissue and bone spectra from very close, close, and clear resection margins. The parameters of bone neogenesis and fibrosis were significantly (*p* < 0.0001) higher in bone-infiltrating tumor tissue than in very close, close, and clear bony resection margins. The parameters of bone neogenesis and fibrosis were significantly (*p* < 0.0001) higher in very close resection margins than in close and clear margins. Bone at close resection margins showed significantly (*p* < 0.0001) higher parameters of bone neogenesis and fibrosis than clear margin bone.

The presence and frequency of lymphocyte and macrophage infiltrates differed significantly (*p* < 0.0001) between bone-infiltrating tumor tissue and bone spectra from very close, close, and clear resection margins. Lymphocytes and macrophage infiltrates were significantly (*p* < 0.0001) higher in bone-infiltrating tumor tissue than in very close, close, and clear bony resection margins. Lymphocytes and macrophage infiltrates were significantly higher in very close resection margins than in close (p = 0.0014) and clear (*p* < 0.0001) margins. Bone at close resection margins contained significantly (p = 0.0090) more lymphocytes and macrophage infiltrates than in clear margin bone (Table 2).

**Table 2:**
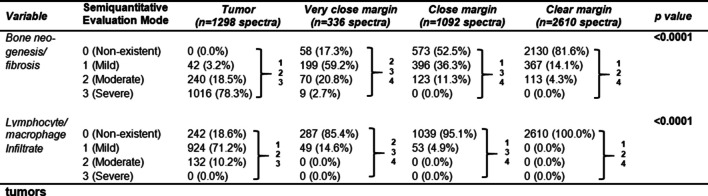
Comparison of histological parameters between very close, close, and clear margins in bone and bone-infiltrating tumors

Histological parameters of chronic osteomyelitis according to Tiemann et al. [1] are indicated as numbers (with percentages). Differences between parameters were analyzed using the Kruskal–Wallis test and with the Dunn’s post hoc test for pairwise testing. Significant p values are bolded. 1: *p* < 0.05 vs. very close margin. 2: *p* < 0.05 vs. close margin. 3: *p* < 0.05 vs. clear margin. 4: *p* < 0.05 vs. tumor

### Dependencies of electrolyte profile changes on resection margin status

Changes in the electrolyte profiles showed significant dependencies from the resection margin status. The transition from very close resection margins to bone-infiltrating tumor tissue made increases in K and decreases in soluble Ca and soluble Ca/K ratio significantly (*p* < 0.001) more likely. The transition from close to very close resection margins made an increase in K (*p* < 0.001) and soluble Ca (p = 0.001) and decrease in the soluble Ca/K ratio (*p* < 0.001) significantly more likely. The transition from clear to close resection margins made increases in K and soluble Ca and decreases in the soluble Ca/K ratio significantly (*p* < 0.001) more likely (Table 3).

**Table 3:** Logistic regression analysis for electrolyte tracking

*Variable*	*Unit*	*OR (95% CI)*	*p value*
*Very Close Margin vs. Tumor*			
*K*	AU	30.488 (17.464–53.223)	** < 0.001**
*Soluble Ca*	AU	0.660 (0.623–0.700)	** < 0.001**
*Soluble Ca/K*	AU	0.639 (0.595–0.686)	** < 0.001**
*Close vs. Very Close Margin*			
*K*	AU	2.148 (1.677–2.752)	** < 0.001**
*Soluble Ca*	AU	1.075 (1.029–1.122)	**0.001**
*Soluble Ca/K*	AU	0.984 (0.978–0.989)	** < 0.001**
*Clear vs. Close Margin*			
*K*	AU	8.018 (6.131–10.486)	** < 0.001**
*Soluble Ca*	AU	1.098 (1.072–1.124)	** < 0.001**
*Soluble Ca/K*	AU	0.985 (0.982–0.987)	** < 0.001**

Data are presented as odds ratio (with p-value) corresponding to logistic regression analysis for prediction of tumor, very close margin, and close margin separately based on K, soluble Ca, and soluble Ca/K ratio with adjustment for age, gender, and G-status. Significant p-values are shown in bold

### Assessment of the tumor border and resection margin status

The transition from very close resection margins to bone-infiltrating tumor tissue can be determined using a cutoff value of ≥ 1.9505 for K with a sensitivity of 95.0% and specificity of 95.5%. The transition from close to very close bony resection margins can be determined using a cutoff value of ≥ 21.7500 for soluble Ca, with a sensitivity of 59.8% and specificity of 62.1%. The transition from clear to close bony resection margins can be determined using a cutoff value of ≥ 0.3015 for K with a sensitivity of 85.3% and specificity of 45.6% (Table 4).

**Table 4:** ROC analysis

*Variable*	*AUC (95% CI)*	*p value*	*Cutoff*	*Sensitivity*	*Specificity*
*Very Close Margin vs. Tumor*					
*K*	0.992 (0.996–0.988)	** < 0.0001**	≥ 1.9505	0.950	0.955
*Soluble Ca*	0.949 (0.939–0.959)	** < 0.0001**	≤ 17.6195	0.827	0.908
*Soluble Ca/K*	0.992 (0.990–0.995)	** < 0.0001**	≤ 10.5785	0.955	0.943
*Close vs. Very Close Margin*					
*K*	0.618 (0.585–0.651)	** < 0.0001**	≥ 0.4725	0.661	0.519
*Soluble Ca*	0.604 (0.569–0.638)	** < 0.0001**	≥ 21.7500	0.598	0.621
*Soluble Ca/K*	0.581 (0.547–0.615)	** < 0.0001**	≤ 37.6005	0.521	0.607
*Clear vs. Close Margin*					
*K*	0.711 (0.694–0.728)	** < 0.0001**	≥ 0.3015	0.853	0.456
*Soluble Ca*	0.496 (0.476–0.516)	0.674	≤ 21.9555	0.653	0.423
*Soluble Ca/K*	0.679 (0.661–0.697)	** < 0.0001**	≤ 64.6055	0.773	0.485

Data presented as area under the curve (AUC), cutoff value, and sensitivity and specificity separately for prediction of resection margin status (very close vs. tumor, close vs. very close, and clear vs. close) based on K, soluble Ca, and soluble Ca/K ratio

## Discussion

Using LIBS as a method for the rapid analysis of bone is intended to address a major challenge in oncology and surgery by allowing intraoperative assessment of native bone. After training tissue recognition algorithms to distinguish bone-infiltrating tumor tissue from healthy bone [25], we recently demonstrated the high potential of LIBS-based depth profiling at the tumor border of bone-invasive oral cancer [26] and characterized the electrolyte heterogeneity of different tumorous and nontumorous tissues in bone-infiltrating oral cancer [27]. The present paper now focuses on the practical and, thus, surgically relevant implications arising from the assessment of bony resection margin status in bone-infiltrating oral cancer with LIBS.

In the current study, the distinction between positive (tumorous) and very close (nontumorous) resection margin LIBS spectra corresponded to the exact and spatially high-resolution visualization of the tumor border. The high sensitivity and specificity in the direct LIBS-guided visualization of the tumor border was primarily based on the high discriminatory power of K emission values. In this context, the current study confirms the results of our proof-of-principle study [25]. Furthermore, the role of K is consistent with the prominent importance of K in our previous LIBS studies on bone-invasive oral cancer diagnostics, in which we detailed the biological basis of the tumor-associated increase in K and its significance in the course of bone-invasive growth in oral cancer [25–27]. Using spectroscopic methods, the tumor boundary in oral cancer could previously only be visualized using indirect methods, such as Raman spectroscopy, whose visualization of the tumor border was based on the increased water content of the tumor tissue compared with oral soft tissue [18]. The ability to visualize the tumor border using direct spectroscopic techniques such as LIBS has already been demonstrated in other tumor entities, such as the brain [20] and skin tumors [22, 23, 31]. The current study is the first to show that the boundary between healthy bone and bone-infiltrating cancer tissue can be determined by LIBS-based measurement of spatial electrolyte distribution.

However, oncological safety is defined not only by the detection of the tumor boundary but also by the accurate determination of resection margins at an adequate distance from the cancer border. Because locoregional recurrence and survival are critically influenced by oral cancer resection margin status [5, 32], the present study has focused on a detailed consideration of the electrolyte profiles of LIBS spectra from bone with very close, close, and clear resection margins. Methodologically, distinguishing tumor-free bone spectra from very close, close, and clear resection margins poses a major challenge for LIBS-based tissue differentiation because our previous LIBS studies depended on stronger macroscopic and microscopic differences in the tissues to be differentiated [25, 27].

In the current study, the significant dependency between the electrolyte profile of K and resection margin status means that the detection of a higher K amount within the bone substance increases the probability of obtaining inadequate and positive resection margins. This finding could biologically indicate that bone, although not yet infiltrated by the tumor, may already be altered by tumor-associated processes. This hypothesis is consistent with the literature because the microarchitecture of cortical bone changes in proximity to oral cavity cancer [33]. In addition, both inflammatory conditions [34] and stromal fibroblasts [35] appear to be involved in promoting bone-invasive cancer growth. Against this background, the chronic HOES criteria (bone neogenesis/fibrosis and lymphocyte/macrophage infiltrates) [28] might be the correlates of the significant differences in very close, close, and clear margin bone LIBS spectra. The microscopic differences between the bone altered in the vicinity of the tumor and the bone from clear margin areas with fatty bone marrow spaces can be detected by the LIBS experimental setup, whose single-shot sensitivity was optimized in preliminary work on the instrumentation of LIBS on natively lasered sample material [24]. As a continuation of our former research efforts, K not only differentiated between healthy bone material and bone-infiltrating cancer tissue [25] and between nontumorous and tumorous tissues in bone-infiltrating oral cavity cancer [27], but it now also differs significantly within the bone substance depending on the distance from the histologically validated tumor border.

Intraoperatively, surgical decision-making could benefit from the ability to predict the bony distance from the tumor. In the present study, the LIBS-based discrimination between bone spectra derived from very close and close resection margins indicates a particularly high susceptibility to incorrect determination of the resection margin status at distances between 1 and 5 mm from the tumor border. In routine surgical oncology, however, it is not the distinction between very close and close margins, but the distinction between close and clear margins with a cutoff value of 5 mm that corresponds to the highest prognostic relevance because the local recurrence rate is 38.2% for close margin resections and 19.4% for clear margin resections [32]. In the present study, LIBS detects the transition from clear to close resection margins with a sensitivity of 85.3%, which could provide intraoperative guidance for estimating the resection margin status at prognostically relevant distances from the tumor border in bone.

Current procedures to prevent positive bone margins and local recurrence in bone-infiltrating oral cancer involve either increasing the surgical radicality to distances of 15 mm from the tumor border [8] or intraoperative sampling of bone scrapings and inferior alveolar nerve tissue [16]. However, because these procedures are based solely on preoperative imaging and assessment of the intraoperative macroscopic situation, there is still an incalculable risk of inadvertently cutting into the tumor tissue, which can lead to tumor cell dissemination with the spread of the tumor cells in the surgical area [36]. In contrast, with ablative laser procedures, the risks of spreading vital tumor cells into the surgical site because of the tissue-consuming effect [37] and outside the surgical site because of the laser smoke [38] appear to be negligible. In addition, LIBS is used as a spectroscopic optical sensor that consumes only a minimal tissue volume of 0.0081 ml with each laser shot [26].

For surgical translation, LIBS could be used intraoperatively before resective osteotomy and as a way to assess the bony resection margin status after resective osteotomy. Considering that LIBS performs a direct measurement on native bone within a few minutes [24–27], LIBS-based visualization of the tumor border and assessment of the bony resection margin distance could represent a significant advantage over the current state of the art, which requires eight days for histological validation by the pathologist [39]. The productive synergism of LIBS-derived depth profiles [26] and high spatial resolution of the tumor border shown in the present study could open up groundbreaking opportunities, as intraoperative detection of the tumor border in three dimensions could enable LIBS-guided resection of highly complex tumors. Based on the results of our preliminary work on LIBS-based rapid bone analysis [24–27] and the present study, the following procedure could be conceivable: Once the border of bone-infiltrating tumors has been precisely defined in all dimensions using LIBS, bone-infiltrating tumors can be resected with a safety margin of > 5 mm as measured from the LIBS-determined tumor border. Subsequently, the margins of the resection specimen can be examined using LIBS again. As shown in our proof-of-principle study [25] and in the present study, LIBS-based tissue detection algorithms could detect positive resection margins in bone with high sensitivity and specificity. If the bony resection margins are tumor free, LIBS can provide indicative information on whether a resection too close to the tumor border has taken place. Because this algorithm preserves the possibility of intraoperative reresection in bone, LIBS could increase the chance of realizing a clear margin resection in bone, whose entire resection surface can be completely captured as opposed to intraoperative sampling [16]. In addition, this concept might preserve more functional bone substance compared with bone resections at least 15 mm away from the tumor border [8].

The future potential for improvement lies in the automation of gridding for higher spatial resolution, as shown by Choi et al. [22], in the use of machine learning algorithms, which have been shown to increase the accuracy of tumor delineation [20–22], and in increasing the number of LIBS spectra. In the future, our results need to be validated on vital tissues.

## Conclusion

LIBS is a promising technique for the objective identification of the tumor border and the assessment of the resection margin status in bone-infiltrating oral cavity cancer. Changes in the K electrolyte profile constitute a reliable indicator for defining the tumor border and might provide an orientation for obtaining a clear margin tumor resection.

## Data Availability

The data that support the findings of this study are available from the corresponding author upon reasonable request.
